# Advancements in Machine Learning for Precision Diagnostics and Surgical Interventions in Interconnected Musculoskeletal and Visual Systems

**DOI:** 10.3390/jcm14113669

**Published:** 2025-05-23

**Authors:** Rahul Kumar, Chirag Gowda, Tejas C. Sekhar, Swapna Vaja, Tami Hage, Kyle Sporn, Ethan Waisberg, Joshua Ong, Nasif Zaman, Alireza Tavakkoli

**Affiliations:** 1 Rush Medical College, Rush University Medical Center, Chicago, IL 60612, USA; tejas_sekhar@rush.edu (T.C.S.); swapna_vaja@rush.edu (S.V.); 2 Miller School of Medicine, University of Miami, Miami, FL 33146, USA; gowdachirag24@gmail.com; 3 Department of Biological Sciences, Virginia Tech, Blacksburg, VA 24061, USA; tamimhage@gmail.com; 4 Norton College of Medicine, SUNY Upstate Medical University, Syracuse, NY 13210, USA; spornk@upstate.edu; 5 Department of Clinical Neurosciences, University of Cambridge, Cambridge CB2 0SZ, UK; cambridgemlgroup@gmail.com; 6 Department of Ophthalmology and Visual Sciences, Kellogg Eye Center, University of Michigan, Ann Arbor, MI 48105, USA; ongjo@med.umich.edu; 7 Department of Computer Science and Engineering, University of Nevada, Reno, NV 89557, USA; zaman@nevada.unr.edu (N.Z.); tavakkol@unr.edu (A.T.)

**Keywords:** artificial intelligence, machine learning, convolutional neural networks, optical coherence tomography, musculoskeletal imaging, ocular biomarkers, choroidal thickness, retinal nerve fiber layer, spine diagnostics, degenerative joint disease

## Abstract

Artificial intelligence (AI) is reshaping precision medicine by revealing diagnostic links between ocular biomarkers and systemic musculoskeletal disorders. This review synthesizes clinical evidence on the associations between optical coherence tomography (OCT)-derived parameters, such as retinal nerve fiber layer (RNFL) thinning and choroidal thickness, and conditions including osteoporosis, cervical spine instability, and inflammatory arthritis. The findings, based on an analysis of studies that integrate AI with ocular and musculoskeletal imaging, highlight consistent correlations between ocular microstructural changes and systemic degenerative pathologies. These results suggest that the eye may serve as a non-invasive window into biomechanical dysfunction. This review also discusses the emerging role of AI-assisted surgical systems informed by ocular metrics. Overall, AI-driven ocular analysis offers a promising avenue for early detection and management of musculoskeletal disease, supporting its clinical relevance and interdisciplinary potential.

## 1. Introduction

The human body’s musculoskeletal and visual systems share intricate biomechanical, vascular, and neural pathways historically underexplored in clinical diagnostics. Emerging studies have demonstrated notable correlations between visual disturbances and musculoskeletal dysfunction, particularly in the musculature of the neck and shoulder regions [1,2]. For instance, individuals with age-related macular degeneration (AMD) tend to experience more musculoskeletal discomfort, likely stemming from compensatory postural adjustments and increased strain during visually demanding tasks [1]. One study found strong correlations between visual complaints and musculoskeletal discomfort seen in both AMD and age-matched control patients (*ρ* = 0.60 and 0.59, respectively, *p* < 0.005) [1]. Supporting these findings, a meta-analysis by Sánchez-González et al. (2019) synthesized 21 studies to investigate the relationship between visual systems and musculoskeletal complaints, showing consistent associations between visual system disorders in accommodation and non-strabismic binocular dysfunctions and chronic neck pain and shoulder discomfort [2]. The authors suggest that visual disturbances may alter head posture and visuomotor behavior, exacerbating neck issues and strain. However, they also noted a limitation in the lack of standardized assessment methods across studies, which reduced the statistical power to unify clinical protocols [2].

Building on these foundations, recent advancements in AI, particularly convolutional neural networks (CNNs), have begun to uncover latent connections between ocular biomarkers and systemic musculoskeletal pathologies. These technologies offer a powerful means to uncover non-invasive indicators of systemic pathology, aligning with the aim of this review: to explore and evaluate the diagnostic potential of AI-driven ocular imaging in the context of musculoskeletal disease. For instance, retinal microvascular changes detected via optical coherence tomography (OCT) may have been linked to early-stage osteoporosis [3], while spinal misalignment correlates with optic nerve compression and glaucoma progression [2].

Integrating AI with high-resolution imaging modalities—such as OCT, fundus photography, and MRI—drives the development of predictive models that combine biomechanical, genetic, and proteomic data. These models enhance diagnostic accuracy and guide personalized surgical planning and long-term postoperative rehabilitation. This review evaluates the transformative potential of AI in musculoskeletal and ocular medicine, focusing on validated applications in diagnostics, surgical robotics, and long-term prognostication [1,2,3].

## 2. Methodology

This narrative review was conducted to evaluate the application of artificial intelligence (AI) in linking ocular biomarkers with musculoskeletal disorders. A comprehensive literature search was performed across four major databases: PubMed, IEEE Xplore, Scopus, and ClinicalTrials.gov, covering publications from January 2000 to March 2025. Search terms were developed to reflect the multidisciplinary nature of the topic and included combinations of “artificial intelligence”, “machine learning”, “deep learning”, “convolutional neural networks”, “transformer models”, “ocular imaging”, “retinal biomarkers”, “choroidal thickness”, “optic nerve head morphology”, “osteoporosis”, “cervical spine instability”, “inflammatory arthritis”, “OCT”, “fundus photography”, “MRI”, “digital twins”, “surgical robotics”, and “multi-modal diagnostics”.

To ensure rigor, two independent reviewers conducted the screening process. Titles and abstracts were first screened for relevance, followed by a full-text review. Inclusion criteria were as follows: (1) peer-reviewed studies involving AI applications in ocular or musculoskeletal diagnostics, (2) studies demonstrating clinical validation or translational potential, and (3) studies published in English. Exclusion criteria included non-peer-reviewed literature, animal-only studies without human data relevance, duplicate entries, and articles lacking adequate methodological detail. Discrepancies between reviewers were resolved through consensus or consultation with a third reviewer.

In addition to original research articles, systematic reviews, regulatory white papers, and consensus guidelines were reviewed to contextualize clinical adoption challenges, ethical implications, and model interpretability. Standardized quality assessment tools, including the SANRA (Scale for the Assessment of Narrative Review Articles), were applied to ensure transparency and methodological rigor throughout the review process.

## 3. AI Integration in Musculoskeletal and Ocular Diagnostics as Independent Fields

Integrating AI into musculoskeletal and ocular diagnostics follows a shared methodological framework involving high-resolution data acquisition, algorithmic training, and clinical validation. However, comparative differences in tissue properties, imaging modalities, and disease manifestations necessitate domain-specific strategies for effective implementation. This section outlines the parallels and distinctions in how AI technologies advance each field, while previewing the role of newer AI architectures and learning paradigms that unify both specialties.

### 3.1. Data Acquisition

**Figure 1:**
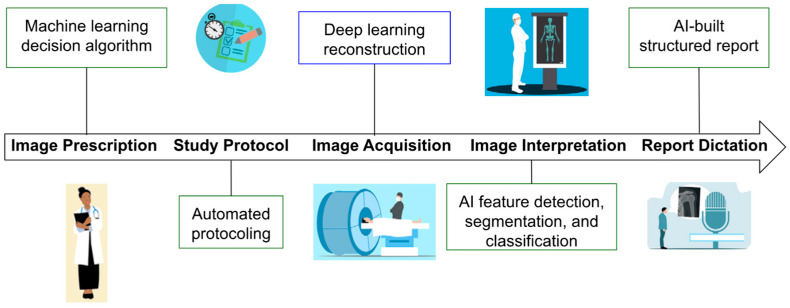
The diagram illustrates the impact of AI on the radiologist’s workflow, highlighting areas of reduced workload (green boxes) and increased workload (blue box). Deep learning-based image reconstruction is expected to raise workload by shortening image acquisition times, enabling more examinations to be conducted within the same time frame. Adapted from Microsoft Word [3].

AI-driven diagnostic workflows in musculoskeletal and ocular care depend on high-resolution imaging modalities, including spectral-domain OCT (SD-OCT), 3-tesla MRI, and musculoskeletal ultrasound (MSK-US) (Figure 1). These technologies generate complex datasets with the granularity needed to train CNNs to detect subtle patterns indicative of systemic pathologies [4,5,6,7].

In ocular imaging, SD-OCT and swept-source OCT (SS-OCT) provide layered retinal scans critical for detecting early degenerative and vascular eye disease. Bellemo et al. demonstrated that CNNs trained on SD-OCT images could detect micrometer-scale deviations in choroidal thickness, which have been associated with musculoskeletal issues such as lumbar disc degeneration [7,8]. Meanwhile, synthetic enhancement of SD-OCT images using generative models has been shown to replicate SS-OCT clarity, improving choroidal visibility in low-resource settings [7,8,9,10,11].

In contrast, musculoskeletal imaging relies more heavily on ultrasound and MRI for dynamic and structural assessment. For example, Clarius MSK AI employs U-Net-based models for real-time tendon segmentation, achieving a Dice coefficient of 0.89 and reducing inter-observer variability in synovitis grading and soft tissue abnormalities [9,10,12].

However, despite the widespread use of SD-OCT in clinical ophthalmology, its limited penetration depth hampers detailed visualization of the choroid, a vascular layer critical for diagnosing chorioretinal diseases. Swept-source OCT (SS-OCT; PLEX Elite 9000, Carl Zeiss Meditec AG, Jena, Germany) overcomes this limitation with enhanced depth imaging but remains costly and less accessible. Bellemo et al. also developed a generative deep learning model that synthetically enhances SD-OCT images (Spectralis OCT, Heidelberg Engineering GmbH, Heidelberg, Germany) to mimic the choroidal clarity of SS-OCT scans. This model was trained on 150,784 paired SD-OCT and SS-OCT images from 735 eyes diagnosed with glaucoma, diabetic retinopathy, or deemed healthy. The AI learned to replicate deep anatomical features, thereby enabling improved visualization of the choroid using standard SD-OCT devices [7,8,10,11].

Performance evaluation using an external test dataset of 37,376 images revealed that clinicians could not distinguish real SS-OCT images from AI-enhanced SD-OCT scans, achieving only 47.5% accuracy, suggesting high realism in the synthetic outputs. Quantitative comparisons further validated the approach: choroidal thickness, area, volume, and vascularity index measurements derived from the AI-enhanced SD-OCT scans showed strong concordance with those from SS-OCT, with Pearson correlation coefficients up to 0.97 and intra-class correlation values as high as 0.99. These findings suggest that deep learning can democratize access to high-quality choroidal imaging, particularly in low-resource settings where SS-OCT is not readily available [7,8,10]. These advancements collectively demonstrate how AI can enhance the quality and accessibility of diagnostic imaging across ophthalmology and orthopedics. While both domains benefit from enhanced spatial resolution and reduced operator variability, ocular imaging is often limited by depth penetration. In contrast, musculoskeletal applications must account for motion artifacts and heterogeneous soft tissue density. These differences guide the choice of imaging modalities and impact AI model design. The evolution of synthetic image generation and enhanced segmentation now sets the stage for advanced model training strategies, including transformer-based architectures and contrastive learning approaches [11].

### 3.2. Algorithm Training

AI systems in ophthalmic and musculoskeletal imaging rely on increasingly sophisticated machine learning (ML) and deep learning (DL) algorithms for tasks such as classification, regression, clustering, and feature extraction [3,13]. Early applications leveraged conventional ML methods—such as logistic regression, decision trees, and nearest-neighbor searches—to identify patterns in radiographic data, including fracture classification and unsupervised lesion clustering [3,11,14]. However, these traditional approaches lacked scalability and spatial awareness, which led to the widespread adoption of CNNs [3,11,14].

**Figure 2:**
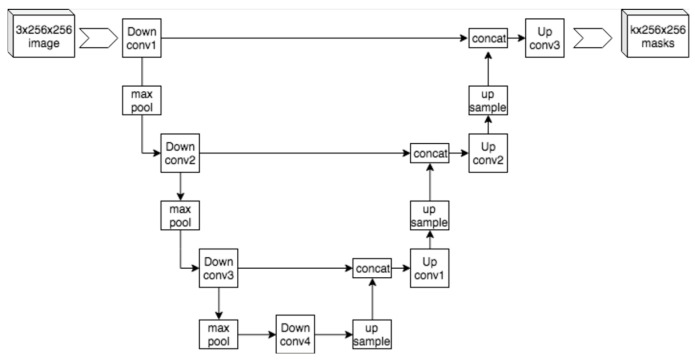
Format of a U-net architecture for producing k 256 × 256 image masks for a 256-by-256 RGB image. This file is licensed under the Creative Commons Attribution-Share Alike 4.0 International license with permission from Wikimedia Commons [11,15,16].

CNNs have become foundational in medical imaging analysis for their ability to extract spatial hierarchies and achieve translational invariance [3,11,14]. In both domains, CNNs are used to segment anatomical structures, detect pathologies, and predict outcomes. A notable advancement in segmentation came with U-Net architectures, which demonstrated high accuracy even with limited annotated data (Figure 2) [11,14,15,16]. Despite their success, CNNs are limited by high data demands, overfitting risk, and interpretability challenges [11,14].

Newer approaches have emerged to address these limitations. Generational adversarial networks (GANs) have been applied to medical image synthesis, domain translation, and resolution enhancement, particularly in synthesizing high-quality musculoskeletal MRI data and enhancing ocular OCT data. However, stability and mode collapse issues persist.

**Figure 3:**
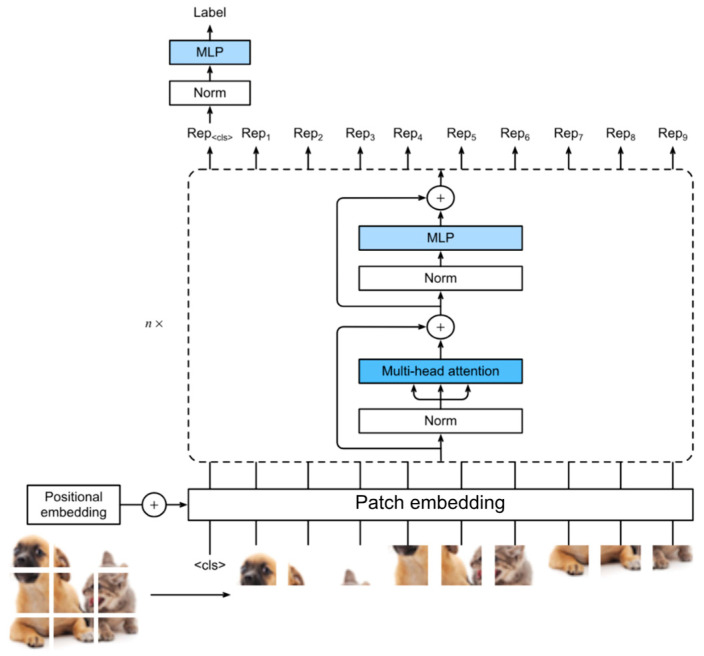
Schematic for vision transformers (ViTs). This file is licensed under the Creative Commons Attribution-Share Alike 4.0 International license with permission from Wikimedia Commons [11,17,18].

Meanwhile, transformer-based architectures offer a compelling alternative by modeling long-range dependencies in data. Vision transformers (ViTs) process images as token sequences, capturing global context more effectively than CNNs (Figure 3) [11,14,17,18]. Swin Transformers enhance this capability by introducing hierarchical attention, making them well suited for volumetric musculoskeletal imaging tasks such as spinal segmentation [11,14,17]. Similarly, the Swin-Poly Transformer, inspired by the Swin Transformer, is specifically designed for OCT image classification, achieving high accuracy and outperforming previous CNN- and ViT-based methods in retinal disease diagnosis [19].

**Figure 4:**
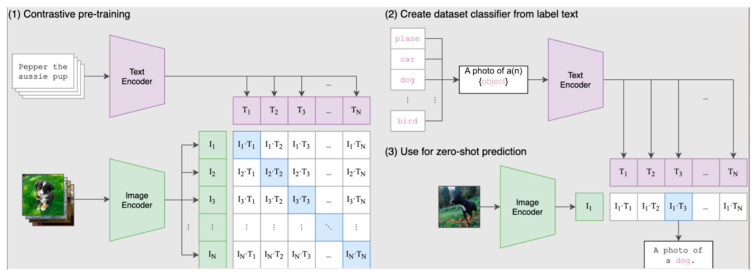
CLIP aligns visual and textual inputs within a shared embedding space, quantified by the Mutual Information (MI) between the two modalities. Visual features extracted by the vision encoder are interpreted through multi-modal concepts, linking object parts to their corresponding textual descriptions. The language encoder identifies textual descriptors near the zero-shot prediction (highlighted in green), shown as surrounding grey points. By comparing the textual representations derived from visual and language encoders, a unified concept space is formed, enabling identification of overlapping descriptors and analysis of shared semantic knowledge across modalities. This file is licensed under the Expat License, sometimes known as the MIT License, with permission granted by Wikimedia Commons. Copyright © The author(s). Permission is hereby granted, free of charge, to any person obtaining a copy of this software and associated documentation files (the “Software”), to deal in the Software without restriction, including without limitation the rights to use, copy, modify, merge, publish, distribute, sublicense, and/or sell copies of the Software, and to permit persons to whom the Software is furnished to do so, subject to the following conditions [24].

**Figure 5:**
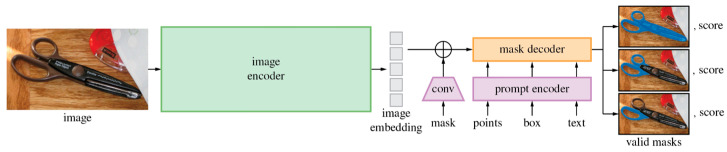
Overview of the Segment Anything Model (SAM) architecture: The SAM utilizes an image encoder to generate image embeddings, a prompt encoder to incorporate user inputs across various prompt types, and a mask decoder that combines these embeddings to produce accurate segmentation masks. The SA-1B Dataset has been released as open source for research purposes. In addition, Meta AI released the pre-trained models (~2.4 GB in size) and permission was granted under Apache 2.0 (a permissive license), following FAIR’s commitment to open research. It is freely accessible on GitHub [25].

Incorporating textual and anatomical labels into training, foundation models such as Contrastive Language–Image Pretraining (CLIP) (Figure 4) and the Segment Anything Model (SAM) (Figure 5) align multi-modal inputs using contrastive learning [20,21,22,23,24,25]. These models enable zero-shot inference and improved generalization, particularly beneficial in ophthalmology, where descriptors like “optic disc pallor” or “macular edema” align closely with visual patterns [22,23,26].

Additionally, diffusion models are gaining traction for their ability to generate high-fidelity synthetic medical images [27]. In musculoskeletal radiology, these models augment datasets for rare pathologies like avascular necrosis, while in ophthalmology, they allow precise simulation of retinal layer abnormalities [27]. Multi-modal learning strategies that fuse imaging, genomic data, and clinical notes further advance model robustness and diagnostic value across both domains [26].

**Figure 6:**
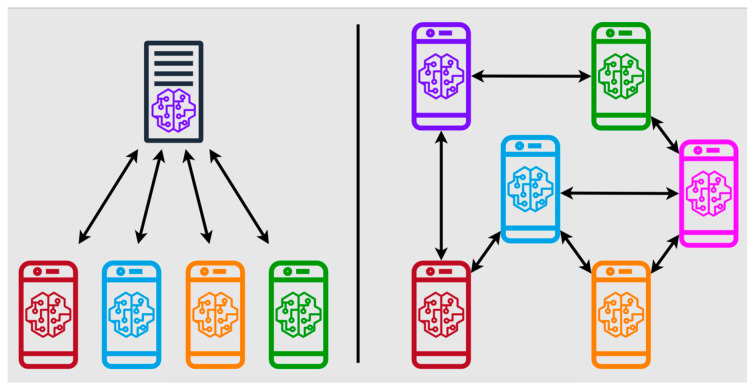
Federated learning (FL) process for medical image analysis, including server and clients. The client trains a model on its local dataset, and the server collects all the models and calculates a global model to train the GANs. This file is licensed under the Creative Commons Attribution-Share Alike 4.0 International license with permission granted by Wikimedia Commons [29,31].

To overcome the challenges of data scarcity and privacy concerns, federated learning frameworks have emerged as key enablers, allowing decentralized training across heterogeneous datasets while preserving patient confidentiality (Figure 6) [11,14,19,28,29]. Quantum machine learning (QML) has evolved with these trends, accelerating model training using quantum processors. One QML prototype reduced training time for musculoskeletal radiomics models by 72% with 54-qubit processors, enabling rapid analysis of T2-weighted MRI scans to predict lumbar disc herniation recurrence [11,30].

Together, these algorithmic innovations drive a shift from static imaging interpretation to intelligent, adaptive diagnostic platforms. Their clinical relevance now hinges on rigorous validation against expert standards—a transition explored in the following section on clinical validation.

### 3.3. Clinical Validation

Comparative clinical validation efforts reveal that AI tools in musculoskeletal diagnostics generally focus on detecting structural pathology, such as fractures, soft tissue injuries, and degenerative disc disease. In contrast, ocular AI applications prioritize vascular and neurodegenerative features, like diabetic macular edema or glaucoma [3,11,14,32].

In musculoskeletal diagnostics, a meta-analysis by Droppelmann et al. reported that 13 AI models achieved 92.6% sensitivity and 90.8% specificity for upper extremity pathologies, outperforming radiologist assessments in multi-center trials [3,11,30]. Similarly, AI models have matched expert-level accuracy in fracture detection [3,11,14,31].

In ocular diagnostics, AI models have successfully identified diabetic macular edema after being trained on fundus photography and OCT images [32]. Other innovations include DL-based ultrasound computer-aided detection and diagnosis (CADe/CADx) systems, which benefit from large-scale ophthalmic image repositories to deliver real-time disease screening in community settings [32].

While musculoskeletal models increasingly integrate radiomic and biomechanical data for prognostication, ocular AI tools are expanding into functional prediction, such as forecasting visual field deterioration or intraocular pressure fluctuations. Both domains now benefit from AI-assisted treatment planning and surgical robotics, underscoring the convergence of diagnostic and interventional AI [3,11,14,31].

This convergence sets the stage for the next section, which explores cross-disciplinary applications and future directions, including AI-driven surgical planning, postoperative outcome prediction, and ethical frameworks for integration in real-world settings.

## 4. AI-Driven Diagnostic Synergies Between Ocular and Musculoskeletal Systems

### 4.1. Convolutional Neural Networks in Multi-Modal Imaging

**Figure 7:**
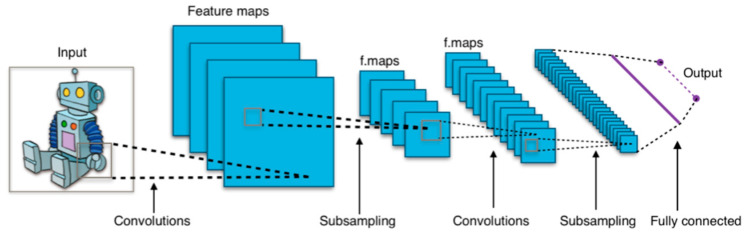
Diagram of a sample convolutional neural network (CNN) architecture designed for binary image classification. The input image passes through five convolutional layers, each followed by a pooling layer that reduces spatial dimensions by a factor of four (from 320 × 320 down to 20 × 20. The convolutional layers extract features at increasing levels of abstraction, while the final fully connected layer handles classification. Output values indicate whether the image contains artifacts or is artifact-free. This file is licensed under the Creative Commons Attribution-Share Alike 4.0 International license with permission from Wikimedia Commons [14,38].

CNNs have become pivotal tools for analyzing spatially structured imaging data across ocular and musculoskeletal modalities (Figure 7). For example, a CNN trained on 8260 knee radiographs achieved 92.3% accuracy in classifying osteoarthritis [33,34,35]. These networks also leverage hierarchical feature extraction in ophthalmology to identify subtle patterns, such as microaneurysms in diabetic retinopathy. These are hypothesized to correlate with accelerated cartilage degradation in osteoarthritis due to shared collagen dysregulation pathways [3,11,36]. Similarly, optical coherence tomography angiography (OCT-A) shows promise in detecting vascular abnormalities associated with musculoskeletal decline [35,36,37].

GANs further enhance diagnostic precision by synthesizing cross-modality images. For example, Spine-GAN segments vertebrae and neural foramina with 94% spatial consistency [3,14,39], while AI-driven OCT analysis quantifies choroidal thickness with submicron resolution, revealing associations with lumbar disc degeneration mediated by impaired glymphatic drainage [6]. Advanced techniques like StarGAN enable synthetic MRI reconstruction from single scans, improving quantitative assessments of musculoskeletal disorders [37,40]. Transfer learning techniques, such as fine-tuning pre-trained ResNet-50 models on retinal fundus images, improve fracture detection sensitivity in upper extremity radiographs [30].

### 4.2. Non-Invasive Ocular Biomarkers for Musculoskeletal Pathologies

Ocular imaging provides a window into systemic health, with AI identifying subtle patterns between ocular biomarkers and systemic musculoskeletal conditions with a high positive predictive value. RNFL thinning, quantified via SD-OCT, predicts cervical spine instability post-whiplash with 91.3% sensitivity and 87.6% specificity [35,37]. Similarly, choroidal thickness variance, measured using enhanced-depth imaging OCT, correlates with lumbar disc degeneration (AUC = 0.82), likely due to shared disruptions in cerebrospinal fluid dynamics [33,37]. In rheumatology, retinal vein tortuosity is lower in ankylosing spondylitis patients than in controls (*β* = 0.1 vs. *β* = 0.5) [11,41]. Simultaneously, Türkcü et al. used OCT-A to reveal reduced vessel density in both the superficial and deep capillary plexuses in psoriasis patients compared to controls, demonstrating systemic inflammatory effects on ocular vasculature [42]. All these studies demonstrate the distinct retinal vessel tortuosity and density patterns across inflammatory musculoskeletal pathologies.

Further supporting the link between ocular imaging and systemic health, Jiang et al. found a significant correlation between reduced bone mineral density (BMD) and decreased choroidal thickness, as assessed by swept-source optical coherence tomography (SS-OCT). In a large SS-OCT study, patients with low bone mineral density (BMD) showed significantly thinner choroidal layers than controls (215.50 μm vs. 229.73 μm, *p* = 0.003) [43,44], supporting hypotheses of shared vitamin D-related and vascular mechanisms [43,44].

Further evidence links ocular findings to neurodegeneration and musculoskeletal decline. In Parkinson’s Disease (PD), reduced choroidal thickness correlates with greater motor symptom severity, higher dopaminergic therapy usage, and increased MRI pathology in the substantia nigra compacta (SNc) [45,46].

In rheumatoid arthritis (RA), CNN-assisted fluorescence optical imaging (FOI) identifies synovitis patterns with 95% concordance with radiologists. In comparison, concurrent OCT scans reveal choroidal thickening in 68% of patients, implicating shared inflammatory pathways [36,44]. Additionally, a study published in Rheumatology (2022) introduced the FOIE-GRAS (Fluorescence Optical Imaging Enhancement-Generated RA Score) system, which confirms these findings and demonstrates high reliability in detecting synovitis in RA patients, with intra-class correlation coefficients ranging from 0.76 to 0.98 [47]. A 2024 systematic review by Fekrazad et al. further confirmed that RA patients exhibited significantly lower choroidal thickness at certain sites than healthy controls, reinforcing ocular imaging’s role in rheumatic disease monitoring [48].

**Figure 8:**
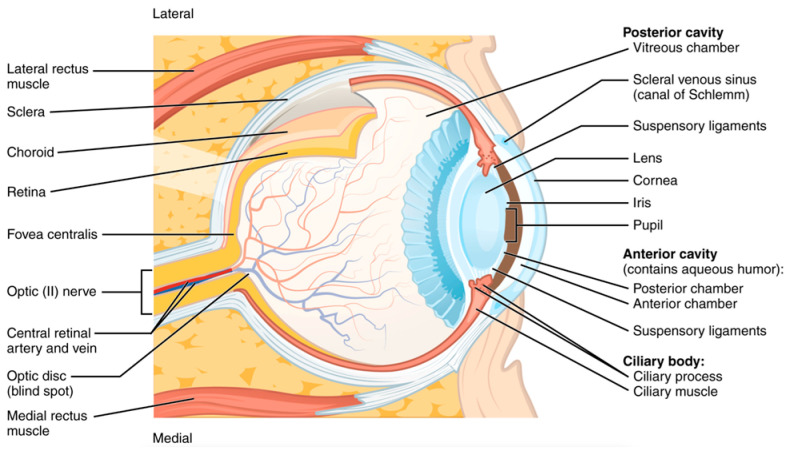
Anatomy of eye and retina. This file is licensed under the Creative Commons Attribution 3.0 Unported license with permission from Wikimedia Commons [49,54].

The retina’s structural and vascular integrity has long been considered a surrogate marker for central nervous system (CNS) health (Figure 8). Ocular imaging has revealed markers of CNS health. RNFL thinning, ganglion cell complex (GCC) loss, and microvascular anomalies in the optic nerve head have been linked to cognitive impairment and Parkinsonian motor dysfunction, which often coexist with musculoskeletal frailty and increased fall risk [49,50,51,52]. AI models trained on OCT data have shown sensitivity exceeding 87% in distinguishing Alzheimer’s patients from healthy controls based on RNFL and macular thickness maps [52,53].

In aging populations, musculoskeletal degeneration is frequently paralleled by declining cognitive and visual function—a phenomenon that AI is helping to quantify through multi-modal diagnostics [55,56]. For instance, conjunctive analysis of OCT-derived GCIPL thickness and MRI-based cortical atrophy can improve risk stratification for falls and vertebral fractures in elderly adults [51,52,53,55].

Moreover, ocular motor dysfunctions such as altered saccadic eye movements, measurable via AI-enhanced infrared tracking systems, have emerged as early indicators of cerebellar degeneration, which affects gait and postural control [51,56]. These biomarkers could be key in preemptively identifying patients at risk of musculoskeletal complications secondary to neurodegeneration [49,50,55]. Collagen profiling via retinal imaging has identified subtype-specific structural vulnerabilities in patients with Ehlers–Danlos syndrome (EDS)—a connective tissue disorder with musculoskeletal and ocular manifestations. Gharbiya et al. reported that ~16% of hypermobile EDS patients exhibited significant retinal atrophy and choroidal thickening. High myopia in hEDS patients was associated with changes in the vitreous extracellular matrix and scleral composition [55,57,58,59]. Another study found that patients with musculocontractural EDS (mcEDS), caused by dermatan sulfate epimerase deficiency, presented with ocular complications such as high refractive errors and retinal detachment [60]. These findings help explain patterns like early-onset joint hypermobility co-occurring with myopic degeneration or retinal detachment [55,56].

Multi-omics integration is expanding this synergy [52,53,55]. RNA-seq from synovial fluid and retinal biopsies reveals overlapping inflammatory signatures in autoimmune diseases such as RA and uveitis [55,57]. This evidence supports the concept of shared proteomic fingerprints across organ systems, enabling cross-disciplinary diagnosis and treatment optimization [52,57]. In RA, AI-driven transcriptomic profiling of synovial tissue has predicted therapy response to standard biological therapies [61,62]. Similarly, single-cell RNA sequencing (scRNA-seq) has been used in ocular disease to characterize intraocular leukocytes in anterior uveitis, identifying unique intraocular immune profiles associated with disease activity [63].

Quantum-enhanced models like Structural Analysis of Gene and protein Expression Signatures (SAGES) combine sequence-based predictions and 3D structural models to analyze expression data, enhancing the understanding of protein evolution and function [61]. While the application of quantum-enhanced models in this context is still in early development, integrating multi-omics data with imaging holds promise for identifying patients at risk for drug-induced musculoskeletal toxicity or predicting the progression from ocular findings to systemic diseases. A summary of all the AI-driven diagnostic findings to elucidate the connection between ocular biomarkers and musculoskeletal disease can be found in Table 1.

### 4.3. AI-Enhanced Surgical Interventions

#### Robotic-Assisted Orthopedic and Ophthalmic Systems

**Figure 9:**
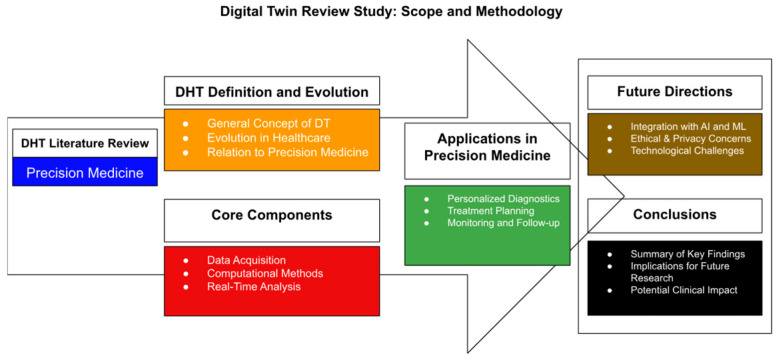
Overview of the applications of digital twins in healthcare. Adapted from Microsoft Word [65].

These diagnostic insights extend into AI-enhanced interventions. Digital twin technology—virtual, AI-enhanced replicas of individual patients—is emerging as a transformative tool in precision medicine (Figure 9). By modeling patient-specific anatomical and biomechanical data, digital twins can simulate disease progression and surgical outcomes in real-time [64,65]. In musculoskeletal care, digital twins have been used to replicate lumbar spine dynamics, predicting outcomes of spinal decompression surgery with 92.1% accuracy [64,65,66,67,68]. These twins integrate MRI, CT, and intraoperative OCT data to refine implant sizing, trajectory planning, and postoperative rehabilitation strategies [68].

Ophthalmology applications are also evolving. Corneal and retinal digital twins are being developed using OCT and adaptive optics data to simulate disease progression in glaucoma and AMD. These platforms allow testing of virtual interventions, such as IOP-lowering surgery or anti-VEGF injection timing, before real-world deployment [68]. The Siemens Healthineers Cardiac Digital Twin program is now being extended to orthopedic and ophthalmic use cases, offering predictive analytics incorporating genetic, proteomic, and radiologic profiles [64,68]. Such simulations enable clinicians to test multiple interventions in silico before entering the operating room [64,65,66,67,68].

AI-driven robotic platforms like Stryker’s Mako system enhance surgical precision by integrating real-time imaging feedback. The Mako system utilizes preoperative CT scans to create patient-specific 3D anatomical models, enabling surgeons to plan and execute procedures accurately. While the Mako system is primarily designed for orthopedic procedures, advancements in intraoperative imaging, such as optical coherence tomography (OCT), have shown promise in improving surgical outcomes in various specialties. For instance, intraoperative 3D imaging has been demonstrated to reduce pedicle screw-related complications and reoperations in spinal fusion surgeries [69,70]. Additionally, OCT angiography (OCT-A) has emerged as a valuable tool in visualizing choroidal neovascularization in diabetic patients, aiding in the assessment and management of diabetic retinopathy [71]. These technologies, when integrated with AI algorithms, have the potential to dynamically adjust surgical trajectories based on live imaging metrics, further enhancing the precision and safety of surgical interventions. 

In joint arthroplasty, AI-enhanced navigation systems achieve submillimeter implant alignment accuracy, with femoral cuts measured within 2° and tibial cuts within 3° of CT-based measurements [65,72,73]. Autonomous surgical robots have shown high accuracy in pedicle screw placement in spine surgeries, with studies reporting accuracy rates up to 99%, resulting in smaller incisions, reduced blood loss, and shorter hospital stays [74,75]. Real-time feedback loops, incorporating intraoperative OCT and inertial measurement units (IMUs), enable adaptive control of robotic end-effectors, minimizing iatrogenic nerve damage during complex spinal revisions [68,69,70,71,72,73,74,75,76].

### 4.4. Postoperative Outcome Prediction

**Figure 10:**
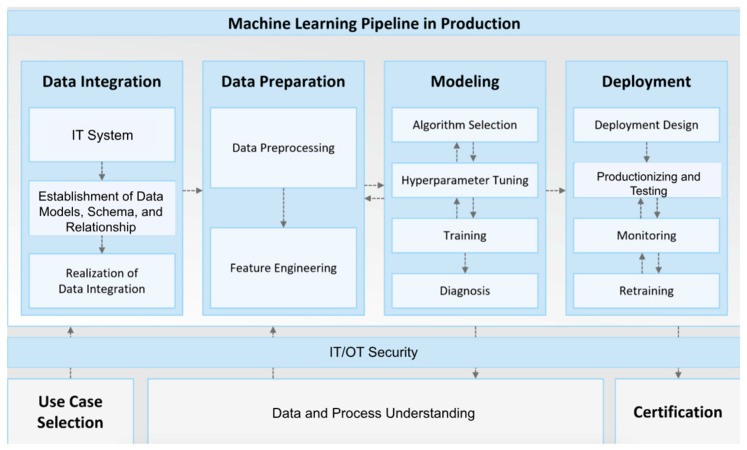
An overview of a proposed machine learning pipeline for production applications. This file is licensed under the Creative Commons Attribution-Share Alike 4.0 International license with permission from Wikimedia Commons [77,85].

CNNs trained on electronic health records (EHRs) and postoperative imaging accurately predict complications. Studies have shown that DL models outperform traditional machine learning methods in predicting postoperative outcomes, utilizing intraoperative physiological data for enhanced prognostication (Figure 10) [77,78,85]. OCT-A has been used to visualize retinal vasculature in conditions like diabetic retinopathy and retinal vascular occlusions, offering quantitative metrics that could potentially correlate with systemic health indicators [79,80].

Predictive models analyzing saccadic eye movements and tear composition accurately forecast delirium risk post-orthopedic surgery, outperforming traditional clinical scores [81,82,83,84].

### 4.5. Translational Research and Clinical Trials

While artificial intelligence continues to redefine diagnostics and surgical precision in musculoskeletal and ocular medicine, its transition from laboratory innovation to clinical routine remains dependent on robust translational frameworks. Multiple prospective clinical trials and real-world implementation studies aim to validate AI-driven diagnostics, predictive modeling, and surgical assistance platforms.

One prominent example is the Bridge2AI Initiative, launched by the U.S. National Institutes of Health (NIH). This initiative funds the development of ethically sourced, multi-modal datasets designed for training clinical-grade AI algorithms. It actively supports cross-disciplinary projects linking ocular imaging biomarkers (e.g., OCT metrics) to systemic pathologies such as osteoporosis and rheumatoid arthritis [80,86].

Similarly, ClinicalTrials.gov lists over 70 ongoing trials involving AI-assisted orthopedic or ophthalmic diagnostics. For example, a prospective study (NCT05369423) evaluates an AI-powered retinal imaging platform for predicting bone density loss and fracture risk in postmenopausal women, which could replace DEXA scans in resource-limited settings [87].

Another study (NCT05921345) investigates the use of AI-enhanced portable OCT devices in mobile musculoskeletal clinics, measuring the effectiveness of RNFL-based biomarkers in predicting spinal degeneration across ethnically diverse populations [88].

In orthopedic surgery, AI-powered navigation tools and robotic arms, such as those used in Stryker’s Mako platform, are clinically evaluated for postoperative complication reduction and precision enhancement [89]. The AI-ROBOT trial, a multi-center initiative in the European Union, compares standard spinal fusion outcomes with those assisted by AI-guided robotic systems (ClinicalTrials.gov ID: NCT05844498) [80,84].

These translational studies are essential for regulatory approvals, clinician adoption, patient trust, and evidence-based reimbursement policies. Embedding explainable AI (XAI) and incorporating human factors analysis into these trials will further ensure that these systems are not only accurate but also usable, interpretable, and scalable [77]. By integrating retinal metrics into AI-driven musculoskeletal models, it may be possible to develop holistic diagnostic systems capable of identifying systemic disease trajectories before overt symptoms appear.

## 5. Emerging Imaging Technologies and AI Integration

### 5.1. High-Field MRI and Photon-Counting CT

Recent advancements in imaging technology have significantly enhanced musculoskeletal diagnostics. The advent of 3-tesla MRI scanners—such as those from Englewood Health—enhances spatial resolution by 40% over conventional 1.5 T systems, enabling visualization of small joint structures like the wrist and ankle with unprecedented detail [77,90,91,92]. Complementing this, photon-counting detector CT (PCCT) reduces radiation doses while improving tissue contrast, facilitating early detection of osteolytic lesions in multiple myeloma [92,93,94]. These advancements synergize with AI algorithms for automated bone age estimation, reducing inter-observer variability from 14.3% to 2.1% in pediatric cohorts [35,80,95].

### 5.2. Next-Generation OCT Systems

Zeiss’s Cirrus 6000 OCT system, with its expanded reference database of 870 healthy eyes, supports data-driven workflows for diagnosing glaucoma and macular degeneration. This comprehensive database enhances individualized diagnostic approaches by accounting for diverse optic disc sizes and age variations [96]. Portable OCT devices, such as the Gen 3 low-cost system, employ balanced detection and quantum-optimized spectrometers to achieve dynamic ranges of 120 dB, rivaling commercial systems [97]. These devices integrate AI for real-time choroidal thickness mapping, detecting osteoporosis risk with 89% accuracy in rural clinics [87,98].

## 6. Challenges and Ethical Considerations in AI-Driven Ocular and Musculoskeletal Diagnostics

Despite the substantial clinical promise of artificial intelligence (AI) in ocular and musculoskeletal diagnostics, widespread implementation is challenged by technical, ethical, and translational concerns. These include data bias, model interpretability, privacy, liability, and resource accessibility—factors that must be addressed to ensure safe, equitable, and effective deployment.

### 6.1. Algorithmic Bias and Data Interoperability

AI model performance is highly dependent on the quality and diversity of training data. Many large-scale ocular imaging datasets disproportionally represent individuals of European ancestry, while underrepresenting African, South Asian, and indigenous populations. This imbalance introduces systemic bias, particularly for diseases like glaucoma or diabetic retinopathy, where presentation differs across ethnic groups [6,99,100]. Similar disparities exist in musculoskeletal imaging, with limited representation of older adults, those with disabilities, and patients from low-resource environments.

Additionally, imaging datasets span heterogeneous formats such as DICOM, JPEG, and proprietary vendor-specific encodings, which complicate their integration. Standardization efforts like the DICOM ophthalmology extensions (WG-09) enable seamless AI training across modalities [35,99]. Due to format variability, AI models may misinterpret or misclassify inputs without such harmonization.

### 6.2. Model Interpretability and Clinical Trust

Despite the promise of artificial intelligence in diagnostics and surgical planning, one of the key hurdles to widespread clinical adoption remains trust among clinicians and patients. A 2025 ISAKOS survey revealed that 47.9% of orthopedic surgeons distrust AI due to limited real-world validation [101]. A critical barrier is DL models’ “black box” nature, particularly convolutional neural networks (CNNs) and transformer architectures. To improve interpretability, explainable AI (XAI) can be integrated into medical platforms to enable clinicians to interpret algorithmic decisions transparently. Methods such as Grad-CAM heatmaps, which weight feature maps using class gradients, and saliency maps have been applied to CNNs analyzing OCT and MRI images, allowing verification of influential diagnostic features. For example, fracture classification models using Grad-CAM have identified periosteal contours and trabecular disruptions, aiding clinician validation [102,103].

These visualization tools also enhance patient–provider communication by tangibly illustrating disease progression. In ophthalmology, differential privacy (DP) techniques, such as pixel-level noise applied to retinal images, maintain diagnostic accuracy within 3% of non-anonymized models while preserving anonymity [104,105]. Nonetheless, interpretability methods vary in robustness and should be evaluated alongside standardized benchmarks and clinician-in-the-loop review workflows.

### 6.3. Privacy and Computational Resource Constraints

To address privacy concerns, federated learning frameworks decentralize data storage, enabling collaborative model training without sharing raw data (e.g., hospital networks training disease detection models locally). However, the computational demands of training 3D CNNs on high-dimensional imaging inputs like OCT volumes remain prohibitive in resource-limited settings where GPU infrastructure may be unavailable [19,81].

### 6.4. Liability and Cross-System Misdiagnosis

As AI is increasingly used to generate diagnostic insights that influence clinical decisions, clear protocols for accountability are essential [106,107]. In cross-system applications—such as predicting spinal degeneration based on retinal thinning—misclassification can result in unnecessary imaging, patient anxiety, or inappropriate interventions [108]. When such errors occur, ambiguity remains around whether responsibility lies with the algorithm developers, health institutions, or the interpreting physician [106,107,108].

To mitigate these risks, governance frameworks must include model audit trails, transparent risk disclosure, and informed consent procedures that acknowledge AI-generated input [107,109]. Regulatory pathways should prioritize clinical validation and involve multidisciplinary oversight [109].

### 6.5. Ethical Frameworks and Global Standards

International organizations have published ethical guidelines to guide responsible AI use in healthcare. The World Health Organization (WHO, 2021) outlines key principles including inclusiveness, accountability, explainability, and data protection [110,111]. Similarly, the European Commission’s Ethics Guidelines for Trustworthy AI emphasize robustness, transparency, and human oversight in clinical applications [112].

Embedding these principles into institutional AI adoption strategies will be vital [111,112]. Moreover, evaluations must go beyond accuracy metrics to consider social impact, particularly equity, usability, and scalability across diverse healthcare environments [111].

### 6.6. Research Gaps and Translational Challenges

Etiological Mechanisms: While complement system dysregulation (e.g., elevated C1qb, C5, and CFH in Modic changes) is linked to disc degeneration, the pathways connecting choroidal thinning to spinal pathologies remain unclear [6,113].

Non-Invasive ICP Monitoring: Current OCT-based ICP estimation lacks validation against invasive measurements, limiting its utility in managing SANS [81,114].

Long-Term AI Efficacy: Prospective trials are needed to validate AI’s role in preventing implant loosening, as current studies rely on retrospective cohorts [101].

## 7. Future Directions: Toward a Unified Framework for Global AI Deployment

The convergence of quantum computing, portable diagnostics, autonomous surgical systems, and global collaborative initiatives signals a paradigm shift in AI-driven medicine—one that is not only technologically advanced but also ethically grounded, equitable, and scalable across health systems. Rather than treating these technologies as isolated innovations, their synergistic integration forms the foundation of a cohesive ecosystem for next-generation diagnostics and therapeutics.

### 7.1. Quantum Computing and Portable Diagnostics

Quantum computing and quantum ML, a novel integration of quantum computing and AI, substantially enhance the processing of large-scale, high-dimensional datasets such as multi-omics data and 3D imaging. These technologies accelerate model training and enhance diagnostic accuracy by identifying subtle associations in complex biological systems [115]. For instance, quantum computing has identified correlations between ocular collagen ratios and tendon elasticity in Ehlers–Danlos syndrome [58,59,60]. By uncovering these associations, quantum-enabled analytics can significantly advance early diagnosis, offering novel biomarkers that facilitate targeted and preventive clinical strategies [116,117]. In surgical applications, quantum computing can empower autonomous robotic systems by enabling real-time optimization of intraoperative decisions, particularly for high-precision tasks in orthopedics and ophthalmology. As surgical robots learn from vast procedural histories, quantum-enhanced feedback loops may minimize error margins, reduce recovery times, and improve outcomes through adaptive control.

### 7.2. Portable AI-Powered Diagnostic Devices

Portable diagnostic devices represent another frontier in healthcare innovation, significantly extending clinical capabilities into underserved regions. Handheld OCT-CNN platforms like the Optovue iWellness device exemplify this advancement, offering clinicians reliable tools for early detection of systemic diseases such as osteoporosis. When coupled with federated learning frameworks, these compact devices can train local AI models while protecting patient privacy by transmitting only encrypted model updates [117]. For example, portable OCT devices deployed in rural clinics across East Africa or South Asia could enhance global datasets and refine retinal biomarker models without compromising data sovereignty.

### 7.3. Autonomous Surgical Systems

Next-generation autonomous robots represent a significant leap in surgical precision and patient healing outcomes. These advanced robotic devices, having learned from over 45 million procedure histories in their databases, can perform intricate surgery with awe-inspiring accuracy, to an unprecedented level of accuracy of 0.2 mm. These technologies amplify surgical procedures such as AC reconstruction, and recovery times have been reduced by an average of 4.3 weeks. The precision is due to the reduced tissue trauma, recovery times, and enhanced patient satisfaction with improved outcomes [117].

Furthermore, AI–human hybrid surgical platforms are emerging that blend autonomous robotics with real-time intraoperative OCT imaging guidance and traditional surgical intuition. This new technology greatly enhances surgical efficacy and safety, with a major reduction in complications related to spinal fusion surgery by some 29% over traditional methods. By integrating robotics capability and human judgment, these hybrid systems optimize surgery procedures for maximum effectiveness, enhance the precision of decision-making during critical moments of surgery, and establish new standards of minimally invasive surgery practices. The combination is set to transform the landscape of surgical procedures with enhanced and safer clinical results [117].

### 7.4. Global Collaborative Initiatives

To support this evolution, international cooperation is required to address dataset variability challenges and facilitate global access. The World Health Organization (WHO), in its 2021 report Ethics and Governance of Artificial Intelligence for Health, emphasizes inclusive, transparent, and equitable AI deployment, particularly in low-resource settings [118,119,120]. These principles are increasingly reflected in practical applications. For example, AI-powered portable imaging devices are deployed in underserved regions to improve healthcare access. In Rwanda, AI-enabled handheld fundus cameras have successfully screened for diabetic retinopathy, improving remote clinics’ referral uptake and diagnostic efficiency [121,122].

Similarly, while data on OCT-A-powered osteoporosis screening in rural South Asia remain limited, AI-based screening tools have demonstrated high accuracy in identifying bone loss in patients with chronic obstructive pulmonary disease, suggesting broader applicability in similar settings [121,122].

### 7.5. Data Harmonization

Furthermore, the NIH’s Bridge2AI and the EU’s AI-ROBOT trials rely on data harmonization, aligning imaging protocols, annotation standards, and metadata structures to ensure interoperability across regions and institutions [123,124]. Initiatives like OpenMRS Version 3.2.1 and the Medical Open Network for AI (MONAI, Version 1.2.0) provide open-source frameworks that allow for local clinical needs without significant financial burden, supporting sustainable and scalable innovation [121,122]. Their flexibility fosters innovation and customization, making them particularly valuable in regions with unaffordable or impractical proprietary solutions. These tools and initiatives exemplify how ethical, collaborative, and open-source approaches can bridge global healthcare disparities through AI [121,122].

Multilingual AI models support global initiatives by interpreting clinical notes, radiology reports, and patient interactions across linguistic boundaries, ensuring that AI-powered platforms remain accessible and inclusive. The AI-MIRACLE Study demonstrates the use of large language models like ChatGPT 4.0 for translating and simplifying radiology reports, helping to overcome prevalent language barriers and improving comprehension in healthcare communication [125].

In summary, quantum computing, portable diagnostics, autonomous robotics, and global collaborations are not siloed advances; they are interdependent components of a unified, global health infrastructure. At their intersection lies the vision of an intelligent, responsive, and universally accessible healthcare system. Realizing this vision will require continuous investment in ethical standards, computational equity, and collaborative innovation.

## 8. Conclusions

AI’s transformative impact on musculoskeletal and ocular medicine lies in its unique ability to decode complex interdependencies between seemingly clinically disparate systems. In ophthalmology, AI algorithms enhance surgical planning and precision in cataract and retinal procedures by analyzing preoperative data and images and streamlining documentation for ocular surface diseases [126]. Similarly, AI-powered image generation technology is reshaping clinical ophthalmic practice by optimizing screening workflows and generating synthetic ophthalmic images, though its adoption requires careful validation to mitigate misuse risks [127].

In parallel, musculoskeletal medicine has seen significant advances in AI-driven triage, detecting fractures (including pediatric cases) and segmenting vertebral and meniscal pathologies with high sensitivity [3]. AI personalizes treatment plans for conditions like osteoarthritis by leveraging ML to recommend tailored interventions [128]. Advanced applications include radiomics for tumor differentiation and prognostication, though clinical translation remains challenging for multi-tissue assessments (e.g., joint MRI) [3,128].

From non-invasive biomarker detection to autonomous surgical robots, AI bridges clinical specialties. Robotics in diagnostics, such as Robotic Ultrasound Systems (RUSSs), standardizes imaging and adapts to patient anatomy using AI, reducing dependency on operator skill [129]. In surgery, autonomous systems like the Smart Tissue Autonomous Robot (STAR) outperform human surgeons in precise tasks like bowel anastomosis [130]. Integration with AR and 3D modeling further refines tumor resection margins in robotic breast surgery, as seen in studies combining MRI-derived models with real-time navigation [131].

Importantly, high-resolution ocular imaging reveals diagnostic insights into systemic diseases. Studies show choroidal thinning correlates with lumbar disc degeneration [6,33], RNFL thinning predicts cervical spine instability with over 90% sensitivity [35,37], and OCT-A-detected microvascular anomalies reflect inflammatory changes in conditions like rheumatoid arthritis and ankylosing spondylitis [41,42,43,44,45,46,47,48]. These findings underscore a paradigm shift: the retina may serve as a window to the brain and a biomarker-rich portal into the bone and spine.

As AI continues integrating with emerging technologies like quantum computing, federated learning, and portable diagnostics, it is poised to redefine the landscape of precision medicine. Realizing this vision will require prospective clinical validation, interdisciplinary collaboration, and a commitment to ethical deployment. Ultimately, AI is not merely a tool, but a catalyst for unifying fragmented diagnostic pathways into a cohesive, system-wide understanding of human health, promising earlier detection, personalized treatment, and more equitable global healthcare delivery.

## Figures and Tables

**Table 1:** Summary of AI-driven diagnostic findings by disease and imaging modality.

Disease/Condition	Imaging Modality	Ocular Biomarker(s)	Musculoskeletal Correlate	Key AI Applications/Findings
Osteoarthritis	OCT, X-ray	Retinal microaneurysm	Cartilage degeneration	CNNs achieved 92.3% accuracy in OA classification; microvascular retinal signs linked to collagen dysregulation [3,11,36]
Lumbar Disc Degeneration	SS-OCT, MRI	Choroidal thinning	Disc dehydration and misalignment	AI-enhanced OCT detected submicron choroidal thinning; correlation with spinal disc changes [6,33,37]
Cervical Spine Instability	SD-OCT	RNFL thinning	Post-whiplash instability	RNFL thinning predicted cervical instability with 91.3% sensitivity and 87.6% specificity [35,37]
Ankylosing Spondylitis (AS)	OCT-A	Reduced retinal vein tortuosity	Inflammatory joint/facet degeneration	β = 0.1 in AS vs. β = 0.5 in controls; indicates systemic vascular involvement [11,41]
Psoriasis/Psoriatic Arthritis	OCT-A	Reduced vessel density (superficial and deep plexus)	Enthesitis and joint inflammation	Demonstrated microvascular compromise in skin and eye correlating with musculoskeletal flare-ups [42]
Rheumatoid Arthritis (RA)	OCT, FOI, FOIE-GRAS	Choroidal thickening, reduced choroidal thickness	Synovitis, joint erosion	CNN and FOIE-GRAS achieved up to 95% concordance with radiologists; choroid metrics linked to RA severity [36,44,47,48]
Parkinson’s Disease (PD)	OCT, MRI	Choroidal thinning, RNFL loss, GCC loss	Muscular rigidity, tremor	Retinal thinning associated with motor severity and SNc MRI pathology [45,46,50]
Alzheimer’s Disease (AD)	OCT	RNFL and macular thinning	Cognitive impairment, fall risk	AI achieved >87% sensitivity in distinguishing AD from controls based on OCT features [52,53]
Ehlers–Danlos Syndrome (EDS)	OCT, Fundus imaging	Retinal atrophy, choroidal thickening, vitreous anomalies	Joint hypermobility, scoliosis	Retinal signs reflect subtype-specific connective tissue degeneration [55,57,58,59,60]
Osteopenia/Low Bone Mineral Density (BMD)	SS-OCT	Reduced choroidal thickness	Generalized bone loss	Mean choroidal thickness: 215.50 μm vs. 229.73 μm in controls (*p* = 0.003); associated with vitamin D deficiency [43,44]
Postoperative Delirium (Orthopedic)	Infrared eye tracking, OCT-A	Saccadic instability, retinal perfusion markers	Delirium, prolonged recovery	AI models outperformed clinical scores in predicting cognitive complications [63,64,65,66,67,68,69,70,71,72,73,74,75,76,77,78,79,80,81,82,83,84]
